# Depth-Sensing Indentation as a Micro- and Nanomechanical Approach to Characterisation of Mechanical Properties of Soft, Biological, and Biomimetic Materials

**DOI:** 10.3390/nano10010015

**Published:** 2019-12-19

**Authors:** Nikolay V. Perepelkin, Feodor M. Borodich, Alexander E. Kovalev, Stanislav N. Gorb

**Affiliations:** 1 School of Engineering, Cardiff University, Cardiff CF24 3AA, UK; 2 College of Aerospace Engineering, Chongqing University, Chongqing 400044, China; 3 Department of Functional Morphology and Biomechanics, Zoological Institute of the University of Kiel, Kiel 24118, Germany

**Keywords:** characterization of materials, depth-sensing indentation, adhesion, the BG method, non-destructive testing

## Abstract

Classical methods of material testing become extremely complicated or impossible at micro-/nanoscale. At the same time, depth-sensing indentation (DSI) can be applied without much change at various length scales. However, interpretation of the DSI data needs to be done carefully, as length-scale dependent effects, such as adhesion, should be taken into account. This review paper is focused on different DSI approaches and factors that can lead to erroneous results, if conventional DSI methods are used for micro-/nanomechanical testing, or testing soft materials. We also review our recent advances in the development of a method that intrinsically takes adhesion effects in DSI into account: the Borodich–Galanov (BG) method, and its extended variant (eBG). The BG/eBG methods can be considered a framework made of the experimental part (DSI by means of spherical indenters), and the data processing part (data fitting based on the mathematical model of the experiment), with such distinctive features as intrinsic model-based account of adhesion, the ability to simultaneously estimate elastic and adhesive properties of materials, and non-destructive nature.

## 1. Introduction

Characterization of properties is a necessary, and one of the most important steps whenever a new material is created or discovered. Biological materials often demonstrate unique and outstanding properties in terms of structural strength, micro- and nanohierarchy, self-organizing and self-healing. Characterization of these properties is a challenging but important task, as properties discovered in biological specimens may lead to the development of new biomimetic and bioinspired materials and structures. The comprehensive review by Meyers et al. [1] provides insight on structure and properties of biological materials, and their various biomimetic counterparts.

Quite often, materials and structures change their mechanical properties in response to changing environment conditions. Changes in properties of biological tissues and cells can indicate various physiological states, including pathological ones. According to Wu et al. [2], “changes in cell and nuclear mechanics are hallmarks of many human diseases, particularly metastatic cancer, cardiovascular disease, inflammation, laminopathies, host-microbe interactions in infectious diseases, and frailty in aging”.

Despite its practical importance, characterization of mechanical properties of soft materials, including biological ones, is a challenging task. Classical methods of material characterization are well developed and standardized for various purposes, such as the determination of Young’s modulus [3,4], Poisson’s ratio [5] or adhesive strength [6]. However, carrying out classical materials testing becomes increasingly difficult when the tested material is very soft, or is available in small quantities. Kang et al. [7] argue that “testing of micro/nanoscale material specimens shares many similarities with conventional, standardized methods… However, as specimen size decreases, testing presents additional challenges… For example, decreasing sample size results in critical challenges in the preparation, handling, and gripping of specimens; in the application of small displacements and forces; in high resolution stress and strain measurements; and in precise control.” The above statement is confirmed by the real-time materials testing inside scanning electron microscopes via nanomanipulation.

For instance, Cao et al. [8,9] carried out nanomechanical tensile testing of a graphene oxide nanosheets using a microelectromechanical system (MEMS) located directly inside an electron microscope. Clearly, it was impossible to create graphene oxide nanospecimens of pre-defined shape. Hence, to interpret the experimental measurements and estimate stress distribution in the specimens, Cao et al. [8] employed molecular dynamics and Finite Element Method (FEM) simulations that took into account the actual shape and thickness of the graphene specimens. The latter papers clearly demonstrate that size effects makes it increasingly difficult to follow conventional macroscopic experimental procedures.

Nevertheless, such experimental technique as depth-sensing indentation, can be applied without much change to various kinds of materials, including soft materials, and at various length scales. Cantilever-based mechanical probing, such as atomic force microscopy, becomes the only available method for elasticity mapping of living specimens at nanometre length scale [10].

Very often biological structures have gradient of material properties in different directions (length, width, depth) down to microscale [11,12]. This fact makes it impossible to use any kinds of tensile tests for characterisation of gradual changes of material properties. At the same time, various kinds of DSI (micro-/nanoindenter, atomic force microscope) allow testing of such biological materials at various length scales, from macroscale [13] up to nanoscale [14].

It is important to note that when it comes to mechanical contact of elastic bodies, both elastic and adhesive properties of contacting materials affect the resulting measurements. Thus, despite DSI itself is done in similar way at various length scales, the interpretation of DSI data should take into account length scale effects, such as adhesion, in many important situations.

We also need to underline the importance of surface roughness and consideration of related changes in real contact area that can affect adhesion. Experiments of Purtov et al. [15] and modelling of the experiments by Pepelyshev et al. [16], employing Galanov’s model of adhesion between rough surfaces [17], showed that consideration of real contact area is a very important factor in adhesive contact problems. This statement is also nicely confirmed by the analysis of nanoscale static friction by Polyakov et al. [18], and various works that demonstrate the influence of roughness on the value of adhesive pull-off force (e.g., [19,20,21]).

Adhesive phenomena become increasingly important, as the size of the contact area decreases. Kendall [22] described the nano-scale world as “he sticky universe”, emphasizing the domination of adhesive interactions, such as van der Waals forces, at this length scale. Inability to take adhesion into account may also lead to erroneous results when DSI of soft specimens is carried out. Kohn and Ebenstein [23] argue that “although adhesion leads to overestimation of the modulus of compliant samples when analyzing nanoindentation data using traditional analysis techniques, most studies of biomaterials have ignored its effects …Compliant, hydrated materials exhibit very different mechanical behaviors relative to mineralized tissues, and require significant modifications to traditional indentation methods to measure accurate modulus values”. It becomes clear that methods of DSI of soft materials and DSI at micro-/nanoscale should take adhesion into account intrinsically and should be based on mathematical models of adhesive contact, rather than non-adhesive ones. It is worth noting that viscoelasticity, which is inherent in many biological materials [24,25], also makes it difficult to use classical contact mechanics models for the estimation of properties by DSI. However, viscoelastic phenomena are not considered in this work.

In this review paper, we focus the discussion on different DSI approaches, their critical assessment, and our recent advances related to the development of a method for simultaneous identification of elastic and adhesive properties of materials and structures based on experimental measurements of DSI: the BG method, and its extended variant: the eBG method. The BG/eBG methods serve the same purpose in DSI with adhesion, as the well-known Oliver–Pharr method [26] in the conventional non-adhesive instrumental indentation: to provide the complete framework that includes experimental technique, data processing algorithm, and the verification of the model used for data processing. As long as the BG method takes into account adhesive effects in DSI, its scope extends beyond the scope of the Oliver–Pharr method, in particular, to the problems of indentation of soft materials, including biological ones, and indentation at micro- and nanoscale.

The paper is organized as follows. In Section 2, conventional DSI techniques are discussed and critically assessed: (i) identification of elastic properties of specimens using sharp pyramidal indenters, and the Oliver-Pharr method in particular; (ii) identification of adhesive properties using pull-off indentation tests by means of spherical indenters. Section 3 and Section 4 are devoted to the description of the BG and eBG methods, correspondingly, including scope, workflow, advantages and limitations. A numerical example based on the use of the eBG method and an asymptotic model of adhesive indentation of a thin elastic coating is presented in Section 4 as well.

## 2. Depth-Sensing Indentation: Conventional Approaches

In this section, we discuss commonly used approaches and techniques of DSI, including critical reviews. In particular, the importance of adhesion in DSI of soft materials is emphasized.

DSI combined with mathematical methods of contact mechanics allows one to determine such properties of the tested materials as reduced elastic contact modulus $E^{*}$ and the work of adhesion *w*. The modulus $E^{*}$ is defined as

(1) $${\left(E^{*}\right)}^{-1}=\left(1-\nu^{2}\right)E^{-1}+\left(1-\nu_{i}^{2}\right){E_{i}}^{-1},$$

where the elastic moduli and Poisson’s ratios of the specimen and the indenter are denoted as *E*, ν, and $E_{i}$ and $\nu_{i}$ respectively. The quantity $E^{*}$ arises in the Hertz contact theory as the proportionality coefficient, among others, between contact pressure and the total approach of two contacting elastic half-spaces assumed to have paraboloidal shapes [27]. Quite often it is possible to assume the indenter to be a rigid body ($E_{i}=\infty$), hence $E^{*}$ depends only on the properties of the specimen $E^{*}=E/\left(1\;-\;\nu^{2}\right)$). The work of adhesion *w* can be defined as the amount of work, per unit area, required to separate two contacting surfaces to infinite distance [27]. See also discussion in [28] related to the thermodynamic interpretation of this quantity. Practical ways to experimentally estimate the work of adhesion are considered further in the paper.

In commonly used DSI approaches these two quantities, $E^{*}$ and *w*, are identified independently, using two different tests involving nominally sharp pyramidal and smooth spherical indenters respectively. These techniques are discussed below.

### 2.1. DSI by Sharp Indenters: The BASh Formula and the Oliver–Pharr Approach

To the best of our knowledge, Kalei [29] was the first researcher who introduced DSI as an experimental technique. In the early years of the existence of this method, it was common to use sharp pyramidal indenters, e.g., developed by M. Khrushchov and E. Berkovich [30] and later named “Berkovich indenters”.

Although we use the term “sharp indenter”, the reader should realise that nominally sharp indenters (at macroscale) are blunt at nanoscale. For example, Borodich et al. [31] argued that the the indenter shape near the tip can be well approximated by a monomial (power-law) functions of radius. If degree of the monomial d=1 then this is mathematically sharp indenter (cone or pyramid), while if the degree d=2 then this may be interpreted as a sphere or an elliptical paraboloid. They demonstrated that shape of real indenters they considered may vary between 1 and 2. However, more often the degree *d* is close to 2 (see e.g., Kindrachuk et al. [32]), hence it is customary to describe the indenter bluntness by the radius of its spherical approximation. One has to realise that the tip angle of a nanoindenter is much greater than the angle of an AFM tip, therefore the geometrically linear formulation of contact problem may be applied to AFM tests only for very shallow indentation depth.

Shortly after the invention of DSI, it would become clear that the unloading branch of the force-displacement curve (the P−δ curve) could be approximately described using the classical Hertz contact theory. Bulychev et al. [33] suggested to modify known exact formulae of axisymmetric contact for that purpose. In particular, they noticed that the slope of the force-displacement curve for a spherical indenter satisfies the relation

(2) $$S=\frac{dP}{d\delta }=2E^{*}a$$

where *a* is the contact radius.

The expression (2) was then re-written in the following form that is often referred to as the Bulychev–Alekhin–Shorshorov (BASh) relation [34]):

(3) $$S=\frac{dP}{d\delta }=2E^{*}\sqrt{A/\pi }.$$

In the latter formula, the expression $\sqrt{A/\pi }$ replaces the contact radius *a* (*A* is the current contact area). The resulting formula therefore becomes an approximate relation, rather than the exact one (2). However, this allowed material scientists community to apply the BASh relation (3) to the case of widely used pyramidal indenters. The latter fact made the expression (3) the corner stone of commonly used techniques of instrumental indentation, including the one described by Oliver and Pharr (OP) [26], which has become de facto the industrial standard [35].

Indeed, the approximate nature of formula (3) makes it complicate to properly process and interpret indentation results. For instance, Oliver and Pharr [26] had to use rather complex approximate relation between the contact area and the indentation depth δ

(4) $$A=24.5\delta^{2}+C_{0}\delta +C_{1}\delta^{1/2}+C_{2}\delta^{1/4}+C_{3}\delta^{1/8}+\ldots +C_{n}\delta^{1/2^{n}}$$

where “the lead term describes a perfect Berkovich indenter; the others describe deviations from the Berkovich geometry due to blunting at the tip” [26]. Complications also arise from the “pile-up” or “sink-in” of the material around the indenter [36]. Critical discussion related to the OP method can be found in a number of papers, such as [37,38,39,40].

In the recent years, possible improvements to the BASh formula have been suggested by means of correction factors, e.g., [41]:

(5) $$\frac{dP}{d\delta }=\beta_{1}\beta_{2}\beta_{3}\frac{2}{\sqrt{\pi }}E^{*}\sqrt{A},$$

where $\beta_{1}\ldots \beta_{3}$ are correction factors taking into account various aspects of elastoplastic deformation and friction in the contact zone between the indenter and the specimen. The factor $\beta_{1}$ is introduced due to the concept of the effective indenter shape (the Galanov effect) [42,43], $\beta_{2}$ is the factor related to non-circular shape of the indenter imprint, and the factor $\beta_{3}$ is introduced due to the effects of friction between the indenter and the specimen [37,44]. In a recent work, however, Galanov and Dub [39] showed that it is not correct to take the Galanov effect into account by simply using the correction factor $\beta_{1}$. They argued that neglecting the true distance between the indenter and the deformed surface of the specimen lead to wrong estimation in the problem governing parameter δ (indentation depth). Therefore, straightforward application of the OP approach may lead to certain inaccuracies in estimations of the reduced contact modulus $E^{*}$.

Previously, we tested properties of insect adhesive structures [11], insect joints [12], insect cuticle [45,46], snake skin [47,48], gecko setae [49], human teeth [50], mollusk teeth [51], and plant materials [45] using nanoindentation by either Berkovich indenter or AFM tip. However, from practical point of view, the use of nominally sharp indenters sometimes may not be desired, e.g., due to the destructive nature of such a method. What is even more important, is that the above technique does not take adhesion into account which may lead to errors when nanoscale measurements are considered.

### 2.2. Adhesion in Depth-Sensing Indentation, and the Conventional Use of Spherical Indenters

The presence of adhesion in the DSI experiment is twofold. The first aspect is that DSI can be specifically used to study adhesion of different materials [52,53,54,55,56]. Previously, we tested mechanical properties of insect adhesive pads [24,57] and other insect structures containing rubber-like protein resilin [25] using microindentation by spherical indenter tip. It is also very useful approach to measure adhesive properties of biological [58] and biomimetic [59,60] materials. The classic theories of adhesive frictionless contact, including the Johnson-Kendall-Roberts (JKR) [61], Derjaguin-Muller-Toporov (DMT) [62] and Maugis (see e.g., [28]) theories, are well developed and can be readily used for the above purposes.

Most often, the identification of the work of adhesion *w* is based on the use of direct methods [54,55]. One of the most popular approaches is based on the JKR model and direct experimental measurements of the adherence pull-off force $P_{adh}$ between a sphere of radius *R* and an a specimen considered an elastic half-space. The JKR theory readily provides relation between the work of adhesion *w* and $P_{adh}$ in the following form:

(6) $$w=-\frac{2}{3}\frac{P_{adh}}{\pi R}.$$

However, there is evidence that direct measurement of the pull-off force may have poor reproducibility, as the tensile (adhesive) part of the force-displacement readings may be influenced by surface quality. Among the factors that influence pull-off force readings researchers point out surface roughness of the specimen [21] (see also [63]), quality of the probe, e.g., wear, the presence of adsorbed chemicals on the probe surface. These factors become increasingly important at nanoscale, in case of DSI done by means of atomic force microscopy (AFM). In such conditions, one needs to carry out a large number of tests to estimate *w* properly using (6) or a similar method.

The other reason to consider DSI an adhesive contact problem is that adhesion greatly influences the results of the DSI experiment at micro-/nanoscale [64], or when it comes to the investigation of properties of soft and biological materials. Disregarding adhesion in these experiments may lead to significant errors in experimental results. Other researchers support this point too, e.g., Kohn and Ebenstein [23] quoted earlier in the Introduction. While some researchers [23,65] develop modifications to the conventional DSI methods involving sharp indenters, it looks more natural to build the complete methodology around an approach that takes adhesion into account intrinsically. In the following sections we describe a method based on this core concept: the BG method, and its extended variant.

## 3. The BG Method

In 2008, Borodich and Galanov developed a new method (the BG method [64,66,67,68]), which constituted a two-stage framework: (a) experimental stage (DSI by spherical indenters), and (b) data processing stage (fitting the experimental data with an appropriate mathematical model of the DSI test with subsequent verification). Such a workflow allows one to intrinsically take adhesion into account, and evaluate both elastic and adhesive properties of the specimen from a single experiment, not two separate ones, as it is done in conventional DSI discussed above. In this Section we consider the scope and detailed workflow of the original BG method, while its extensions are considered further in the text.

The scope of the original BG method is simultaneous determination of the reduced elastic modulus $E^{*}$ and the work of adhesion *w* in the framework of the classical theories of adhesive contact, like the JKR or DMT. In order to apply these theories, the specimen is considered an elastic half-space.

The workflow of the method begins with depth-sensing indentation by a spherical indenter which results in *N* measurements of the indentation depth $\delta_{i}$ and indentation force $P_{i}$ during DSI: $\left(\delta_{i},P_{i}\right),\;i=1\ldots N$. The basic idea of the method is to find the unknown material properties by taking a mathematical model of the same experiment, that is, the equation that theoretically describes the force-displacement relation, and adjusting control parameters of the model so that the theoretical force-displacement curve best fits the data in certain sense.

In the beginning, the force-displacement dependency is written in a dimensionless form. For example, the governing equations of both the JKR and DMT theories can be represented as

(7) $$F\left(\frac{P}{P_{c}},\frac{\delta }{\delta_{c}}\right)=0.$$

Note the characteristic scales (scaling parameters) of the problem $P_{c}$ and $\delta_{c}$. They are introduced to formulate the force-displacement curve in the dimensionless form. In the BG method, these quantities are subject to adjustment, while the theoretical force-displacement curve (7) is fit to the experimental data points as the result of an optimization process.

Technically, to determine two unknown quantities, $P_{c}$ and $\delta_{c}$, one would need only two equations. Hence, if all the experimental readings were exact, the following identities would be all true at the same time

(8) $$F\left(\frac{P_{i}}{P_{c}},\frac{\delta_{i}}{\delta_{c}}\right)=0,\;i=1,\ldots ,N.$$

In that case, one would just need to select any two of them and solve for $P_{c}$ and $\delta_{c}$.

In the real experiment, however, measuremets $\left(\delta_{i},P_{i}\right)$ always contain certain inaccuracies. Therefore, in general case, the relations (8) are never true all at the same time.

However, one may consider (8) an overdetermined system of equations. Instead of making all the expressions exact at the same time, it is possible to minimize the measure of the total error produced in the system. Consider the quantity

(9) $$\varepsilon_{i}=F\left(\frac{P_{i}}{P_{c}},\frac{\delta_{i}}{\delta_{c}}\right)$$

which is the individual residual of *i*-th equation. If one minimizes the mean square residual $e=\frac{1}{N}\sum_{i=1}^{N}\varepsilon_{i}^{2}$ of the overdetermined system (8), it necessarily leads to the following optimization problem:

(10) $$\left\{P_{c}^{*},\delta_{c}^{*}\right\}=\arg\;\min\;\Phi \left(P_{c},\delta_{c}\right)$$

(11) $$\Phi \left(P_{c},\delta_{c}\right)=\sum_{i=1}^{N}{\left[F\left(\frac{P_{i}}{P_{c}},\frac{\delta_{i}}{\delta_{c}}\right)\right]}^{2}.$$

Here $P_{c}^{*},\delta_{c}^{*}$ are the optimal values of the characteristic parameters that minimize the objective functional Φ and provide best fit of the experimental data. Clearly, the objective functional is the mean square residual multiplied by the number of data points *N*, which is constant.

Methods of solution of such problems are well developed (see e.g., [69,70]). As long as the optimal values of the characteristic parameters $P_{c}=P_{c}^{*}$ and $\delta_{c}=\delta_{c}^{*}$ are found, the sought material parameters $E^{*}$ and *w* can be readily evaluated from them, because the characteristic parameters are known expressions that are defined within the framework of the chosen theory of adhesive contact.

The particular representation of the theoretical curve (7) and the characteristic scales can be different, dependidng on the theory of adhesive contact used to describe the DSI test. For example, in the JKR theory, the characteristic scales may be taken as

(12) $$P_{c}=\frac{3}{2}\pi wR,\;\delta_{c}=\frac{3}{4}{\left(\frac{\pi^{2}w^{2}R}{E^{*2}}\right)}^{1/3}$$

when a spherical indenter of radius *R* is considered. The particular look of the theoretical force-displacement curve (7) in this case is

(13) $$F\left(\frac{P}{P_{c}},\frac{\delta }{\delta_{c}}\right)=\left\{\begin{array}{c} \left(3\chi -1\right){\left((1+\chi )/9\right)}^{\frac{1}{3}}-\delta /\delta_{c}=0,\;\left(\chi \geq 0,\;\delta /\delta_{c}\geq -3^{-2/3}\right),\\ \left(3\chi +1\right){\left((1-\chi )/9\right)}^{\frac{1}{3}}-\delta /\delta_{c}=0,\;\left(2/3\geq \chi \geq 0,\;-3^{-2/3}>\delta /\delta_{c}\geq -1\right) \end{array}\right.$$

where $\chi =\sqrt{1+P/P_{c}}$ [28].

In the JKR theory, $P_{c}$ defines the maximum of the absolute value of the pull-off force, and $\delta_{c}$ is the absolute value of minimum possible displacement among all points representing the P−δ curve. The material properties $E^{*}$ and *w* can be found from Equation (12) as

(14) $$w=\frac{2P_{c}}{3\pi R},\;E^{*}=\frac{P_{c}}{4}\sqrt{\frac{3}{R\delta_{c}^{3}}}.$$

The DMT theory can be used in the BG method too [67]. In the DMT theory, the force-displacement curve can be written in the form [28]

(15) $$F\left(\frac{P}{P_{c}},\frac{\delta }{\delta_{c}}\right)=\frac{P}{P_{c}}-\frac{1}{\sqrt{3}}{\left(\frac{\delta }{\delta_{c}}\right)}^{3/2}+\frac{4}{3}=0$$

with the characteristic parameters defined above.

For the case of the DMT theory, Borodich et al. [67] showed that the evaluation of $P_{c}$ and $\delta_{c}$ can be separated from one another by presenting the corresponding overdetermined system (8) as

(16) $$3^{1/3}{\left(\frac{P_{i}}{P_{c}}+\frac{4}{3}\right)}^{2/3}-\frac{\delta_{i}}{\delta_{c}}=0,\;i=1,\ldots ,N,$$

and re-writing it in the form

(17) $$\begin{array}{c} a_{i}P_{c}-b_{i}=0, \end{array}$$

(18) $$\begin{array}{c} c_{i}\left(P_{c}\right)\delta_{c}-\delta_{i}=0, \end{array}$$

where expressions $a_{i}$ and $b_{i}$ solely depend on the experimental data, and the expression $c_{i}$ depends on both experimental data and $P_{c}$.

The final step of processing experimental data in the BG method is the validation of the initially chosen theory of adhesive contact, and the corresponding mathematical model (7). Clearly, a given data set can be fitted with multiple theoretical force-displacement curves that belong to different theories of adhesive contact. For this reason, the BG method has a verification step: calculation of the Tabor–Muller parameter introduced in the works of Tabor [71] and Muller [72] (see also discussion in [28]). The purpose of this dimensional parameter is to provide clear distinction of applicability range between the JKR and DMT theories of adhesive contact:

(19) $$\mu ={\left(\frac{Rw^{2}}{E^{*2}z_{0}^{3}}\right)}^{1/3}.$$

Here, *R* is the effective curvature radius of contacting bodies, and $z_{0}$ is the equilibrium distance between atoms of the contacting bodies, normally assumed to be 0.3…0.5 nm. The case μ≫1 is normally referred to the JKR theory, while values μ≪1 suggest to use the DMT theory. If the calculated value of μ does not correspond to the theory of adhesive contact that was used in the above calculations, then a different theory should be chosen, and data fitting repeated.

Note that other authors have also used data fitting-related techniques in studies of soft and biological materials. See, for instance, the works [73,74,75,76,77,78]. However, in most cases one can hardly find any detailed description of fitting methods and procedures. Performance-related aspects in terms of accuracy and robustness are omitted too. Only few authors (e.g., [79,80,81]) devote the whole study to the development of methodology of identification of material properties. With this regard, we should note that the above mentioned works [64,66,67,68] contain detailed analysis of the mathematical aspects of the BG method, and its accuracy, including the ability to withstand the presence of measurement noise. The recent work by Perepelkin et al. [82] contains detailed discussion and performance assessment with regards to the extended BG method, which is discussed in the next Section.

## 4. The Extended BG (eBG) Method

In contact mechanics of adhesive contact, only solutions of the simplest, classical contact theories, like JKR or DMT, can be represented as explicit functions. Overall, it is typical to represent the solutions of more complex contact problems in the form of parametric functions, in which both the external load and the indenter displacement depend on the contact radius as the parameter [34]. Among numerous examples of this kind, one can find asymptotic mathematical models of JKR-type contact for layered and coated medium [83,84,85,86], implementations of the Maugis theory [87,88], or the double-Hertz theory [89]. These models have complex mathematical form of parametric functions that cannot be exactly reduced to explicit or implicit ones. In addition, it is fundamentally impossible to do such reduction to semi-analytical models based on the finite elements method calculations [90,91,92].

In its initial formulation, the BG method cannot utilize mathematical models which describe force-displacement curve as a parametric function. The focus of the original method is on the use of the classical theories of adhesive contact. However, this issue has recently been addressed, as Perepelkin et al. [93] announced the eBG method, an extended version of the original approach which has distinctive new features, such as orthogonal distance fitting concept, and two stage fitting approach. Theoretical aspects of the eBG method are presented in the recent work [82], which describes two different implementations of the eBG method. Here we briefly discuss the variant of the eBG method which was successfully used in [93].

The scope of the eBG method is determination of elastic moduli (such as reduced contact modulus $E^{*}$) and the work of adhesion *w* of elastic structures, for which the force-displacement DSI curve can be described as a parametric function:

(20) $$\left\{\begin{array}{c} \delta =\delta_{c}f_{1}\left(\bar{a},\delta_{c},P_{c}\right),\\ P=P_{c}f_{2}\left(\bar{a},\delta_{c},P_{c}\right). \end{array}\right.$$

In these expressions, $\bar{a}$ is the dimensionless parameter, which may have physical interpretation, e.g., dimensionless contact radius.

Normally, fitting a parametric curve requires one to find the values ${\bar{a}}_{i}$ corresponding to each data point from the experimental data $\left(\delta_{i},P_{i}\right),\;i=1\ldots N$. In turn, this increases the number of unknown quantities to the order of thousands, whilst only two quantities, $P_{c}$ and $\delta_{c}$, are the matter of interest for the researcher. To avoid this issue, we introduced [82,93] a two-stage data fitting process.

**Figure 1:**
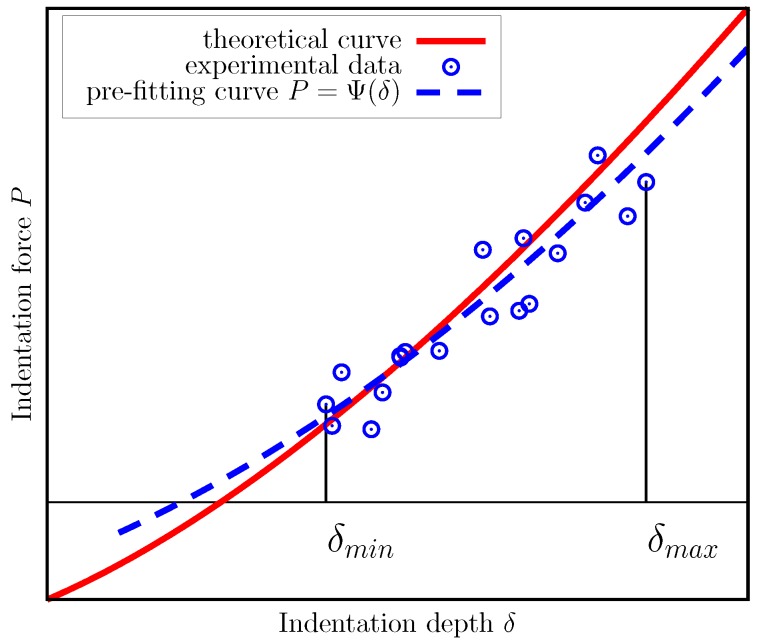
Preliminary fitting the experimental data with an auxiliary curve $\Psi \left(\delta \right)$.

The first stage can be considered data filtering, as typically experimental data contains measurement noise. It is supposed to first smooth the experimental data by fitting it with some auxiliary curve $P=\Psi \left(\delta \right)$ (Figure 1). To make fitting relatively fast and simple process, the mathematical form of $\Psi \left(\delta \right)$ should be rather simple.

If the auxiliary fitting curve (the pre-fitting curve) has simple mathematical form, then advanced fitting techniques can be implemented. In our works, we built such auxiliary fitting curves that best fit experimental data in terms of minimal averaged value of squared orthogonal distance from the data points to the curve (the orthogonal distance curve fitting (ODF) approach [94,95]). The benefit of this approach is that it filters out noise present in both experimental measurements, $\delta_{i}$ and $P_{i}$, whereas the commonly used least squares fitting minimizes residual related to only one measured quantity, either $\delta_{i}$ or $P_{i}$.

Orthogonal distance curve fitting problems are known to be computationally intensive [94,95], hence preliminary fitting (pre-fitting) was done using a polygonal chain (a piecewise-linear function) with the number of segments $N_{S}$, which could vary.

On the second stage the values of $P_{c}$ and $\delta_{c}$ become subject to adjustment, so that the theoretical curve becomes closest to the auxiliary one. This is achieved by minimizing the squared norm of difference of the two functions:

(21) $$\hat{\Phi }\left(P_{c},\delta_{c}\right)=\int_{\delta_{min}}^{\delta_{max}}{\left[P\left(\delta \right)-\Psi \left(\delta \right)\right]}^{2}d\delta .$$

(22) $$\left\{P_{c}^{*},\delta_{c}^{*}\right\}=\arg\;\min\;\hat{\Phi }\left(P_{c},\delta_{c}\right)$$

The optimal values of the scaling parameters, $P_{c}^{*},\delta_{c}^{*}$, are further used to evaluate unknown material properties as discussed in the previous section.

The actual force-displacement curve is supposed to have parametric form (20), therefore, substitution of (20) yields the final form of the objective functional of the eBG method $\hat{\Phi }$:

(23) $$\hat{\Phi }\left(P_{c},\delta_{c}\right)=\delta_{c}\int_{{\bar{a}}_{min}}^{{\bar{a}}_{max}}{\left[P_{c}f_{2}\left(\bar{a},\delta_{c},P_{c}\right)-\Psi \left(\delta_{c}f_{1}\left(\bar{a},\delta_{c},P_{c}\right)\right)\right]}^{2}\frac{\partial f_{1}\left(\bar{a},\delta_{c},P_{c}\right)}{\partial \bar{a}}d\bar{a}.$$

In this functional, $\bar{a}$ is the integration variable. Hence, the optimization problem has dimension two, and the need to find the corresponding value of the parameter $\bar{a}$ for every data point is therefore eliminated.

The above two-stage approach has been experimentally validated in [93] by conducting two independent experiments: (a) a DSI experiment with subsequent use of the eBG method, and (b) a tensile testing of dumbbell specimens made of the same material. The values of the reduced elastic modulus obtained form DSI data by means of the eBG method, and the ones obtained from the tensile testing were in good agreement.

**Figure 2:**
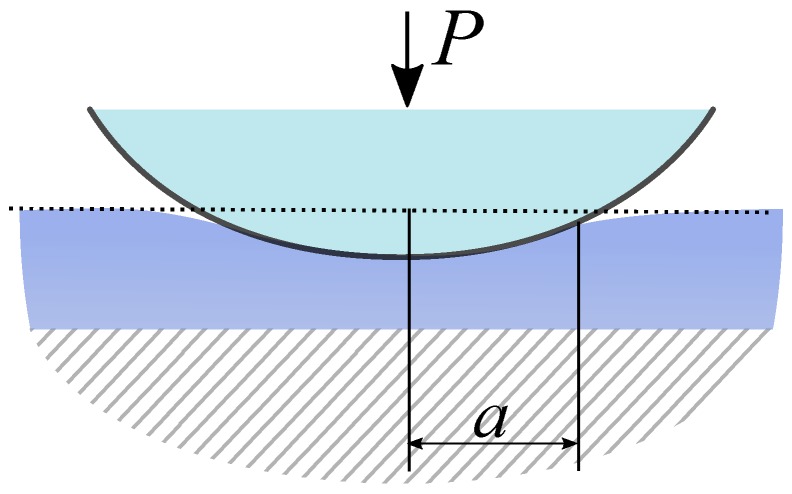
Indentation of a thin elastic layer bonded to the rigid base.

The work [82] contains a number of numerical benchmarks related to the speed and accuracy of the variant of the eBG method shown above. In this paper, however, we present a short numerical example, in which DSI of a thin elastic layer bonded to a rigid substrate is simulated (Figure 2). The indenter is supposed to be a sphere of a known radius *R*.

**Figure 3:**
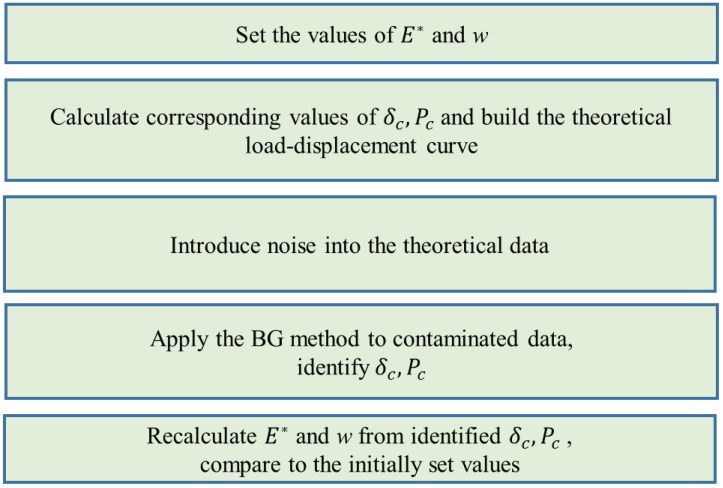
The workflow of the numerical simulation demonstrating the accuracy and robustness of the extended Borodich–Galanov (eBG) method.

The mathematical model of such a DSI experiment in the framework of the JKR theory of adhesive contact can be written in a dimensionless form [85]:

(24) $$\begin{array}{c} \frac{P}{P_{c}}={\bar{a}}^{4}-2{\bar{a}}^{2},\\ \frac{\delta }{\delta_{c}}={\bar{a}}^{2}-1, \end{array}$$

where the characteristic parameters are

(25) $$P_{c}=2\pi Rw,\;\delta_{c}=\sqrt{\frac{2w}{K}},$$

and the model parameters are as follows: $K=E\left(1-\nu \right)/[h\left(1+\nu \right)\left(1-2\nu \right)]$ is the stiffness of an equivalent elastic foundation substituting the layer, *h* is the layer thickness, *E* and ν are the Young’s modulus and Poisson’s ratio of the layer, respectively. The indenter is supposed to be rigid, therefore the reduced contact modulus of the layer is $E^{*}=E/\left(1-\nu^{2}\right)$. The simulation workflow is shown in Figure 3.

**Figure 4:**
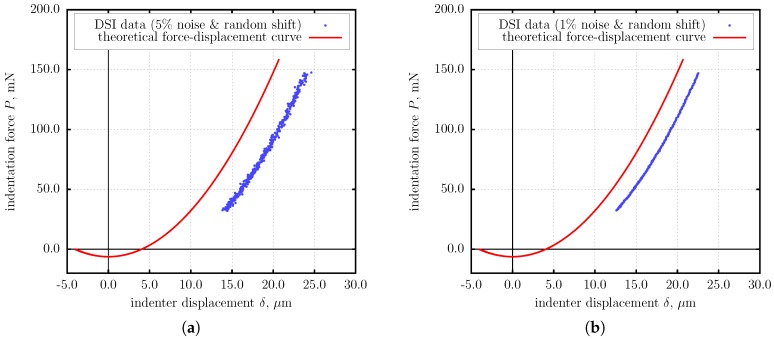
Numerical simulation. The theoretical force-displacement curve (solid line) and an example data sets simulating DSI readings (dots, shifted). (**a**) High noise scenario. (**b**) Low noise scenario.

The simulated model parameters were: E=0.91 MPa, ν=0.3, that is $E^{*}=1$ MPa; R=10 mm, h=0.1 mm, w=100 mJ/m^2^. In the simulation, the Tabor–Muller parameter was calculated after setting all the parameters. Hence, the validity of the JKR theory in this scenario was confirmed a priori, and no further checks were done. The theoretical force-displacement curve is depicted if Figure 4 as the solid line. The compressive part of it was used to generate noisy data emulating a DSI experiment. The noisy data is denoted by points in Figure 4 (shifted).

The values of the indentation depth δ were shifted by a random quantity emulating the uncertainty in the determination of the true indenter position, as the exact distance from the indenter to the point of contact is never known in the real experiment. This shift value was determined as follows. A series of possible shift values was generated. For each shift value minimization of (23) was performed using the modified data set, as the shift value was subtracted from the initially given values $\delta_{i}\;\left(i=1,\ldots ,N\right)$. The absolute minimum of values of the objective functional among all trial minimizations was identified, and the corresponding shift value was supposed to be the true one. Further, the values of $P_{c}$ and $\delta_{c}$ found in the corresponding trial problem were supposed to be the true ones. The issue of uncertainty in the indenter position is discussed in detail in [82].

Two cases were considered in the simulation: (i) high noise scenario (Figure 4a), and (ii) low noise scenario (Figure 4b). In each scenario 20 data sets were generated using normally distributed noise. The eBG method, as shown above, was applied to each data set separately. In each individual case, data points were pre-fitted with a seven-segment polygonal chain using the orthogonal distance fitting approach.

**Figure 5:**
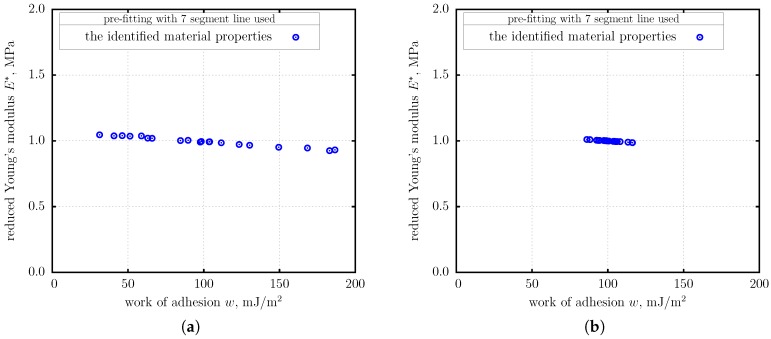
Numerical simulation. The results of identification of material properties from 20 data sets containing noise and random coordinate origin shift. (**a**) High noise scenario. (**b**) Low noise scenario.

The individual identified values of $E^{*}$ and *w* are depicted graphically in Figure 5. It is remarkable, that despite significant spread in the individual identified values, the averaged values of $E^{*}$ and *w* were determined with high accuracy: $E^{*}=0.9948$ MPa and w=99.38 mJ/m^2^ in the high noise scenario, $E^{*}=0.9987$ MPa and w=101.2 mJ/m^2^ in the low noise scenario.

This example demonstrates an important feature of the BG/eBG method: the ability to extract unknown material properties, including adhesive ones, from the stable compressive part of the force-displacement data, in contrast to the methods based on the measurement of the pull-off force, which use less stable tensile part of the DSI data.

## 5. Conclusions

As specimen size decreases, classical methods of material testing become extremely complicated and challenging. At the same time, such experimental technique as depth-sensing indentation (DSI) can be applied without much change at macro-, micro-, and nanoscale. Interpretation of the DSI data needs to be done carefully though, as length-scale dependent effects, such as adhesion, should be taken into account.

In this work, we review our recent advances in the development of a method that intrinsically takes adhesion in DSI into account: the BG method, and its extended variant, the eBG method. The BG/eBG methods can be considered a framework made of the experimental part (DSI by means of spherical indenters), and the data processing part (data fitting based on the mathematical model of the experiment).

The main advantages and distinctive features of the BG/eBG methods are: (i) intrinsic account of adhesion, in contrast to the common methods of instrumental indentation (e.g., the Oliver–Pharr method). Hence, they can be used for determination of adhesive properties in the first instance, or whenever adhesion during DSI cannot be neglected (i.e., micro/nanoindentation, and indentation of soft solids); (ii) the ability to use only stable compressive DSI data to identify unknown material properties, including adhesive ones; (iii) the ability to estimate both elastic and adhesive properties of the specimen in a single experiment, instead of two separate ones, as it is usually done; (iv) the BG method is a non-destructive technique. This provides benefits in terms of: (a) the ability to test living biological samples (e.g., cell membranes); (b) the ability to do repetitive tests at exactly the same location (e.g., to do statistical averaging of the results, or to investigate surface physics under changing environment conditions); (c) non-destructive control of the quality and properties of protective coatings.
